# Altered Brain Function and Causal Connectivity Induced by Repetitive Transcranial Magnetic Stimulation Treatment for Major Depressive Disorder

**DOI:** 10.3389/fnins.2022.855483

**Published:** 2022-03-14

**Authors:** Muzhen Guan, Zhongheng Wang, Yanru Shi, Yuanjun Xie, Zhujing Ma, Zirong Liu, Junchang Liu, Xinyu Gao, Qingrong Tan, Huaning Wang

**Affiliations:** ^1^Department of Mental Health, Xi’an Medical University, Xi’an, China; ^2^Deptartment of Psychiatry, Xijing Hospital, Air Force Medical University, Xi’an, China; ^3^Department of Radiology, Xijing Hospital, Fourth Military Medical University, Xi’an, China; ^4^Deptartment of Psychology, Air Force Medical University, Xi’an, China; ^5^Deptartment of Psychiatry, Yulin Fifth Hospital, Yuling, China

**Keywords:** major depressive disorder, amplitude of the low-frequency fluctuation, regional homogeneity, Granger causality analysis (GCA), repetitive transcranial magnetic stimulation

## Abstract

**Objective:**

Repetitive transcranial magnetic stimulation (rTMS) can effectively improve depression symptoms in patients with major depressive disorder (MDD); however, its mechanism of action remains obscure. This study explored the neuralimaging mechanisms of rTMS in improving depression symptoms in patients with MDD.

**Methods:**

In this study, MDD patients with first-episode, drug-naive (*n* = 29) and healthy controls (*n* = 33) were enrolled. Depression symptoms before and after rTMS treatment were assessed using the Hamilton Depression Rating Scale (HAMD-17). Resting-state functional magnetic resonance imaging (rs-fMRI) data were collected both before and after the treatment. Changes in the brain function after the treatment were compared using the following two indices: the amplitude of the low-frequency fluctuation (ALFF) and regional homogeneity (ReHo), which are sensitive for evaluating spontaneous neuronal activity. The brain region with synchronous changes was selected as the seed point, and the differences in the causal connectivity between the seed point and whole brain before and after rTMS treatment were investigated via Granger causality analysis (GCA).

**Results:**

Before treatment, patients with MDD had significantly lower ALFF in the left superior frontal gyrus (*p* < 0.01), higher ALFF in the left middle frontal gyrus and left precuneus (*p* < 0.01), and lower ReHo in the left middle frontal and left middle occipital gyri (*p* < 0.01) than the values observed in healthy controls. After the rTMS treatment, the ALFF was significantly increased in the left superior frontal gyrus (*p* < 0.01) and decreased in the left middle frontal gyrus and left precuneus (*p* < 0.01). Furthermore, ReHo was significantly increased in the left middle frontal and left middle occipital gyri (*p* < 0.01) in patients with MDD. Before treatment, GCA using the left middle frontal gyrus (the brain region with synchronous changes) as the seed point revealed a weak bidirectional causal connectivity between the middle and superior frontal gyri as well as a weak causal connectivity from the inferior temporal to the middle frontal gyri. After treatment, these causal connectivities were strengthened. Moreover, the causal connectivity from the inferior temporal gyrus to the middle frontal gyri negatively correlated with the total HAMD-17 score (*r* = −0.443, *p* = 0.021).

**Conclusion:**

rTMS treatment not only improves the local neural activity in the middle frontal gyrus, superior frontal gyrus, and precuneus but also strengthens the bidirectional causal connectivity between the middle and superior frontal gyri and the causal connectivity from the inferior temporal to the middle frontal gyri. Changes in these neuroimaging indices may represent the neural mechanisms underlying rTMS treatment in MDD.

**Clinical Trial Registration:**

This study was registered in the Chinese Clinical Trial Registry (Registration number: ChiCTR1800019761).

## Introduction

Major depressive disorder (MDD) is a mental illness characterized by mood disorders and cognitive dysfunction. By the year 2020, MDD had become the second most disabling illness and a major social and public health concern (Cheng et al., 2018). Blood-oxygen-level-dependent magnetic resonance imaging works based on the principle of detecting changes in the magnetic field properties due to the changes in local metabolism and blood oxygen level after neuronal activity (Tian et al., 2020). This imaging method has been widely used in exploring the pathophysiology of and therapeutic mechanism for MDD (Tateishi et al., 2020). The amplitude of the low-frequency fluctuation (ALFF) in the blood-oxygen-level-dependent signal is used to describe the amplitude of the spontaneous activity of the brain neurons, and larger ALFF indicates greater activity in the brain region. Regional homogeneity (ReHo) refers to the correlation of the synchronous activity in the blood-oxygen-level-dependent signal between a given voxel and its neighboring voxels (Dichter et al., 2015). The ALFF and ReHo can evaluate the spontaneous activity of neurons from different perspectives, and these two indices are complementary to each other (Kong et al., 2017).

Patients with MDD have been reported to exhibit abnormal ALFF and ReHo in the brain function (Yao et al., 2014; Li et al., 2018), A recent study investigated the MDD patients with negative bias and showed significantly higher neuronal activation in the frontal lobe (Gollan et al., 2015; Tadayonnejad et al., 2015). Studies have also indicated increased ReHo in the right postcentral gyrus and increased ALFF in the left triangular part of the inferior frontal gyrus (Xia et al., 2019; Rosenbaum et al., 2020; Liu et al., 2021). Liang et al. (2020) demonstrated that compared with healthy controls, patients with MDD had lower ReHo in the calcarine gyrus, insula, right superior temporal gyrus, left cuneus, right precuneus and right postcentral gyrus as well as lower ALFF in the calcarine gyrus, left cuneus, right inferior parietal cortex, right precuneus, and right postcentral gyrus (Kaiser et al., 2015), indicating that brain dysfunction in these patients affects their emotion regulation and social function.

Medication is the primary treatment modality used for patients with MDD. Antidepressants usually begin to show effects after 2 weeks of intake; hence, such medications (e.g., venlafaxine) should be taken for at least 4 weeks before determining the drug efficacy (Gex-Fabry et al., 2004; Otte et al., 2016). However, the first-line medication and psychotherapy often fail to show any improvement in the condition of 20–30% of patients with MDD (Loerinc et al., 2015; Maneeton et al., 2020; Cohen et al., 2021), repetitive transcranial magnetic stimulation—a non-invasive and well-tolerated physical therapy (McGirr et al., 2015) in the domain of antidepressant therapy—has been an important treatment choice (Antonenko et al., 2019; Yin et al., 2020). Zheng et al. (2020) collected the functional magnetic resonance imaging (fMRI) data of 15 patients with MDD both before and after repetitive transcranial magnetic stimulation (rTMS) treatment and found an increase in the ALFF in the left dorsolateral prefrontal cortex and left superior frontal gyrus after 2 weeks of treatment; however, they found no correlation between the change in the ALFF and symptoms of depression.

Granger causality analysis (GCA) is a method used for identifying the effective connectivity of the directed functional interactions from time-series data, and it has been widely used in resting-state fMRI studies (Berlim et al., 2014; Gaynes et al., 2014; Yang et al., 2021). Feng et al. (2016) analyzed the data of 23 drug-naive patients with first-onset MDD using GCA. They revealed that these patients had a stronger causal connectivity from the right insula, right putamen, and right caudate nucleus to the cingulate gyrus seed point as well as a weaker causal connectivity from the bilateral dorsolateral prefrontal and left orbitofrontal cortices to the cingulate gyrus seed point than those of the healthy controls (Feng et al., 2016); this indicated the dysfunction of the prefrontal cortex–limbic system in patients with MDD (Ishida et al., 2020).

However, the mechanism through which rTMS improves depression symptoms remains unclear, and few studies have explored the changes in both the ALFF and ReHo of patients with MDD receiving rTMS treatment. In this study, resting-state fMRI (rs-fMRI) was used to evaluate the changes in the abovementioned indices in patients with MDD after rTMS treatment. The brain region with synchronous changes was selected as the seed point, and the causal connectivity between the seed point and whole brain was analyzed via GCA to investigate the mechanism through which rTMS improves the symptoms of depression in patients with MDD.

## Materials and Methods

### Participants

This study included drug-naive patients with first-onset MDD aged 18–45 years who visited the psychiatry outpatient clinic of the First Affiliated Hospital of Air Force Military Medical University, China, from May 2019 to October 2021. The inclusion criteria were as follows: (1) Patients who fulfilled the MDD diagnostic criteria of the Diagnostic and Statistical Manual of Mental Disorders, Fifth Edition, (2) patients with a total score of ≥ 18 points on the Hamilton Depression Rating Scale (HAMD-17), and (3) patients who met the enrollment requirements of the study and provided their informed consent. The exclusion criteria were as follows: (1) patients with severe physical diseases, (2) patients with a history of traumatic brain injury or brain surgery, (3) patients with a history of alcohol or substance abuse, and patients with a history of other mental or nervous system disorders.

Healthy controls matching the characteristics of patients with MDD were also recruited in this study. All participants read and understood the experimental procedures and precautions and signed informed consent forms before the commencement of the study.

### Clinical Evaluation

In this study, the depression symptoms of patients with MDD were evaluated using the Hamilton Depression Rating Scale (HAMD-17) both before and after rTMS treatment (Hamilton, 1967). Higher HAMD-17 scores indicated more severe depression.

### Repetitive Transcranial Magnetic Stimulation Procedure

The repetitive transcranial magnetic stimulator used in this study is a magnetic field stimulator from YIRYIDE Medical (MagPro R30, Dantec Medtronic, Denmark, CCY-IA), and the stimulation coil is a 100 mm figure of eight shaped coil. The treatment was administered at 10 Hz and 110% of the resting motor threshold (RMT) coil. The RMT is the minimum stimulation intensity that can elicit at least 5 motor evoked potential with an amplitude > 50 μV with 10 consecutive stimuli to the patient (Bashir et al., 2019). According to the international 10–20 system, the rTMS treatment target is located at the F3 point of the left dorsolateral prefrontal region, one pulse per second for 10 s, with 10 s interval, consisted of 1,000 pluses. rTMS treatment was provided for 15 min per day on 15 successive days.

### Research Procedures

In the current study, we conducted a single-blind, randomized, controlled study at a general hospital. The randomization program was created using a computer and executed by an investigator who is not involved in the treatment and recruitment of patients. The allocation of patients was screened, applying numbered in the sealed and opaque envelopes. The antidepressant taken by the patients was venlafaxine (H32022135). The doses of venlafaxine prescribed were tailored based on clinical considerations determined by the physicians. The patients taken oral venlafaxine were initiated at a dose of 75 mg/d. Based on response and tolerability, the dose could be titrated upward to 150 mg/d at a 2-week interval. The patient with MDD used HAMD to assess the severity of depressive symptoms before and after the treatment rTMS treatment and completed the fMRI scan.

### Imaging Data Acquire and Preprocessing

The patients underwent scanning within 48 h before the commencement of rTMS treatment and on the day following the end of the treatment course. The healthy controls were only scanned at baseline. Imaging data were acquired on a 3.0 Tesla MRI system with a standard 8-channel head coil (GE Medical Systems, Milwaukee, WI, United States). Functional images were acquired using a gradient echo-planar imaging (EPI) sequence (repetition time, 2,000 ms; echo time, 30 ms; FOV = 240 mm × 240 mm; FA = 90; matrix = 64 × 64; slice thickness, 4.5 mm; 45 axial slices no gap. A total of 210 volumes were collected for a total scan time of 420 s.

We performed image preprocessing using DPABI^1^ and SPM12.^2^ Briefly steps are as follow: (1) The first ten scan volumes were discarded for steady-state magnetization; (2) subsequent images were corrected for temporal differences by slice timing and head motion by alignment; (3) the resulting functional images were spatially normalized to the standard space of the Montreal Neurological Institute (MNI) using an optimum affine transformation and non-linear deformations, and then resampled to 3 mm × 3 mm × 3 mm isotropic voxels. Nuisance signals, including those from Fristo; (4) then all the functional images were smoothed with a 6-mm full-width at half-maximum (FWHM) Gaussian filter; (5) time series linear detrending was conducted to remove low-frequency drifts and high-frequency physiological noise.

### Amplitude of the Low-Frequency Fluctuation Analysis

The whole-brain ALFF was calculated using the DPABI software. First, the time series of a given voxel was extracted; next, the amplitude of all frequencies in a frequency range (which was set as 0.01–0.1 Hz in this study) was calculated via Fourier transform; then, it was converted to the power spectrum for radication to determine the ALFF. The ALFF of each voxel was standardized (subtracted from the mean value of the whole-brain signal and then divided by the standard deviation) to obtain the standardized ALFF for each participant, which was used in subsequent statistical analyses.

### Regional Homogeneity Analysis

By calculating Kendall’s coefficient of concordance of each voxel with a total of 26 voxel time series adjacent to the point, line, and plane of the given voxel, ReHo was determined and then divided by the mean value of the whole-brain signal to get the standardized ReHo; next, the standardized ReHo was subjected to Gaussian smoothing with the full width at a half-maximum of 6 × 6 × 6 mm^3^ and then used for subsequent statistical analyses.

### Granger Causality Model

In this study, the left middle frontal gyrus with synchronous changes was used as the seed point, and dual-coefficient GCA was used to evaluate the causal connectivity between the time series describing the seed point and that of each voxel in the whole brain. GCA based on the whole-brain voxel level was used in the representational state transfer toolkit; the mean time series of the left middle frontal gyrus was defined as the seed-based time series x, whereas the time series of each voxel in the whole brain was represented by the time series y. A positive value of the causal connectivity from x to y indicated the existence of a causal connectivity between the activity in the middle frontal gyrus and that in the brain in the same direction, whereas a negative value indicated the opposite direction. The seed point was analyzed on the causal connectivity from the seed point to the other voxels in the whole brain (x to y) and that from the other voxels in the whole brain to the seed point (y to x). Afterward, the *Fisher R-to-Z* transformation was performed to obtain the voxel-level connectivity Z-map for subsequent statistical analyses.

### Statistical Analysis

Statistical analysis of the clinical data was performed using SPSS 22.0. The paired-samples *t*-test was used to compare the ALFF and ReHo of patients with MDD before and after the treatment, whereas the independent-samples *t*-test was used to compare these neuroimaging indices of patients with MDD before and after the treatment with those of the healthy controls. The results of multiple comparisons were corrected using a Gaussian random field. The cluster level *p* < 0.01 (false discovery rate correction), voxel level *p* < 0.001, and voxel number > 70 were used to identify the brain region that differed significantly between groups. The imaging indices of different brain regions were extracted and their correlations with HAMD-17 scores were analyzed. *p* < 0.05 was considered statistically significant.

## Results

### Participant Demographics

A total of 29 patients with MDD, with a mean age of 28.44 ± 7.909 years and mean MDD duration of 4.05 ± 3.77 months were included in the treatment group, whereas a total of 33 healthy controls with a mean age of 26.53 ± 5.563 years were included in the control group. There were no significant differences between the two groups in terms of age, sex, and education level (*p* > 0.05; Table 1).

**TABLE 1:** Group demographics and clinical measures (mean ± *SD*).

	MDD (*n* = 29)	Healthy controls (*n* = 33)	*p*
Age (years)	28.44 ± 7.91	26.53 ± 5.56	0.52
Female/male	20/9	22/11	0.88
Education (years)	14.71 ± 2.01	15.18 ± 1.99	0.36
Duration of illness (months)	4.05 ± 3.77	-	
Dose of venlafaxine (mg/d)	98.27 ± 22.16	-	

### HAMD-17 Score

The HAMD-17 total score was significantly higher in patients with MDD than in healthy controls, and the total score after treatment was significantly lower in patients with MDD than that before treatment in (*t* = 11.313, *p* < 0.001; Table 2).

**TABLE 2:** Comparisons of the score of HAMD-17 between MDD and healthy controls (mean ± *SD*).

	MDD	Healthy controls	*t*
Pre-rTMS	21.00 ± 5.382	2.85 ± 2.279	18.571***
Post-rTMS	9.52 ± 4.106	-	7.961***

*** means p < 0.001.

### Amplitude of the Low-Frequency Fluctuation Analysis

**FIGURE 1:**
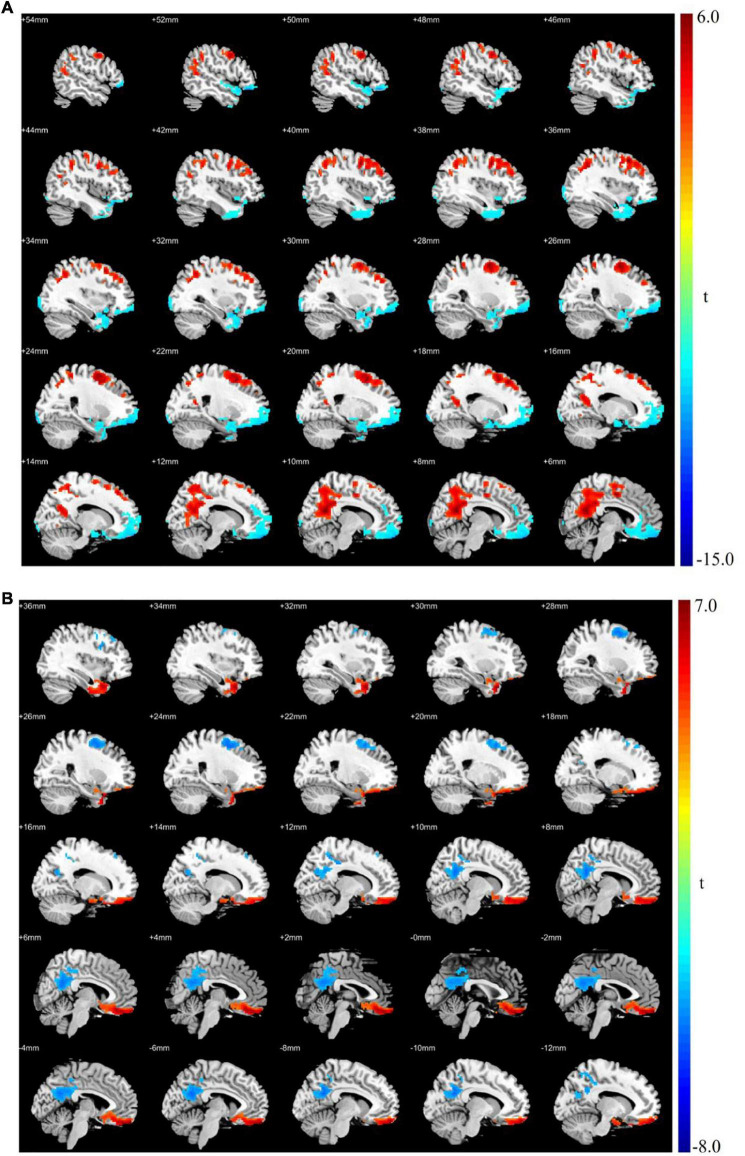
**(A)** Brain regions showing significant differences of amplitude of low-frequency fluctuation (ALFF) between MDD and healthy controls. **(B)** Brain regions showing significant differences of ALFF between pre- and post-rTMS. The warm color denoted the region where ALFF is higher, and the cool color denotes the region where ALFF is lower.

Patients with MDD had lower ALFF in the left superior frontal, right inferior temporal, left middle occipital, and right inferior occipital gyri as well as higher ALFF in the left precuneus and left middle frontal gyrus than the values observed in healthy controls (Table 3 and Figure 1).

**TABLE 3:** Brain regions in ALFF between MDD and healthy controls.

Brain regions	Brodmann areas	Side	MNI coordinates			Cluster size	*t-*values
			*X*	*Y*	*Z*		
Superior frontal gyrus	11/10	Left	−12	60	−21	133	−8.651
Inferior temporal gyrus	20	Right	69	−27	−27	76	−6.483
Middle occipital gyrus	17	Left	−27	−102	3	207	−7.827
Inferior occipital gyrus	18	Right	30	−99	−15	79	−7.421
Precuneus	40	Left	−42	−42	36	370	6.711
Middle frontal gyrus	8	Left	−36	12	60	313	6.619

After 15 days of rTMS treatment, the ALFF was increased in the left superior frontal gyrus and decreased in the left precuneus and left middle frontal gyrus of patients with MDD (Table 4 and Figure 1).

**TABLE 4:** Brain regions in ALFF between post- and pre-rTMS.

Brain regions	Brodmann areas	Side	MNI coordinates			Cluster size	*t*-values
			*X*	*Y*	*Z*		
Superior frontal gyrus	11	Left	−12	57	−21	115	8.5434
Precuneus	23	Left	−6	−57	24	141	−5.859
Middle frontal gyrus	44	Left	−48	12	36	99	−5.747

### Regional Homogeneity Analysis

**FIGURE 2:**
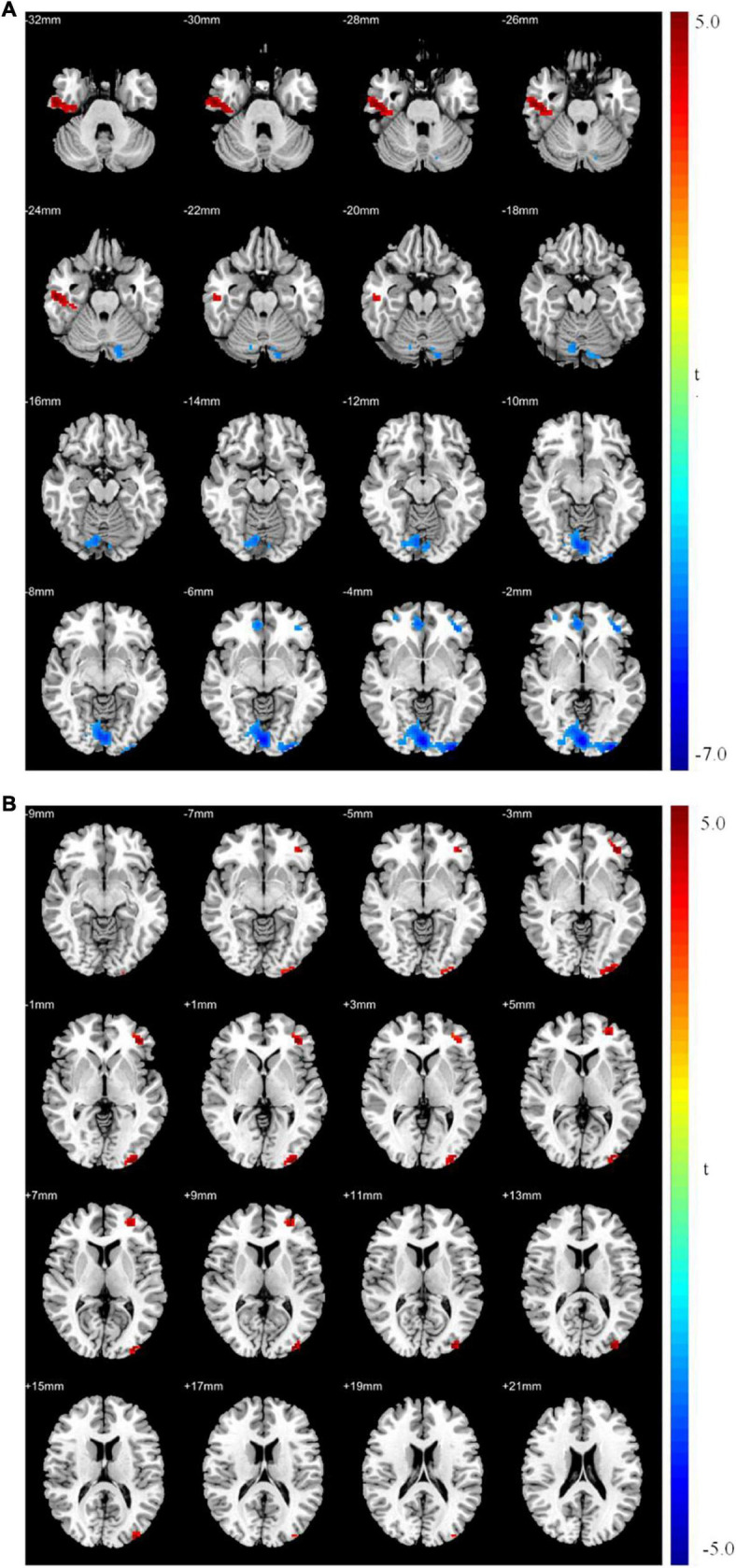
**(A)** Brain regions showing significant differences of regional homogeneity (ReHo) between MDD and healthy controls. **(B)** Brain regions showing significant differences of ReHo between pre- and post-rTMS. The warm color denoted the region where ReHo is higher, and the cool color denotes the region where ReHo is lower.

Patients with MDD had higher resting-state ReHo in the right inferior temporal gyrus and lower ReHo in the left middle occipital, left middle frontal, and left postcentral gyri than the values obtained in healthy controls (Table 5 and Figure 2).

**TABLE 5:** Brain regions in ReHo between MDD and healthy controls.

Brain regions	Brodmann areas	Side	MNI coordinates			Cluster size	*t*-values
			*X*	*Y*	*Z*		
Inferior temporal gyrus	20	Right	54	−18	−24	164	4.851
Middle occipital gyrus	17	Left	0	−90	−3	488	−7.469
Middle frontal gyrus	24	Left	−34	39	15	87	−5.282
Postcentrol gyrus	3	Left	−57	−21	45	62	−5.8024

After 15 days of rTMS treatment, ReHo was increased in the left middle occipital and frontal gyri (Table 6 and Figure 2).

**TABLE 6:** Brain regions in ReHo between post- and pre-rTMS.

Brain regions	Brodmann areas	Side	MNI coordinates			Cluster size	*t*-values
			*X*	*Y*	*Z*		
Middle occipital gyrus	18	Left	−33	−96	0	74	4.189
Middle frontal gyrus	47	Left	−39	39	−3	57	4.237

### Granger Causality Analysis Results

**FIGURE 3:**
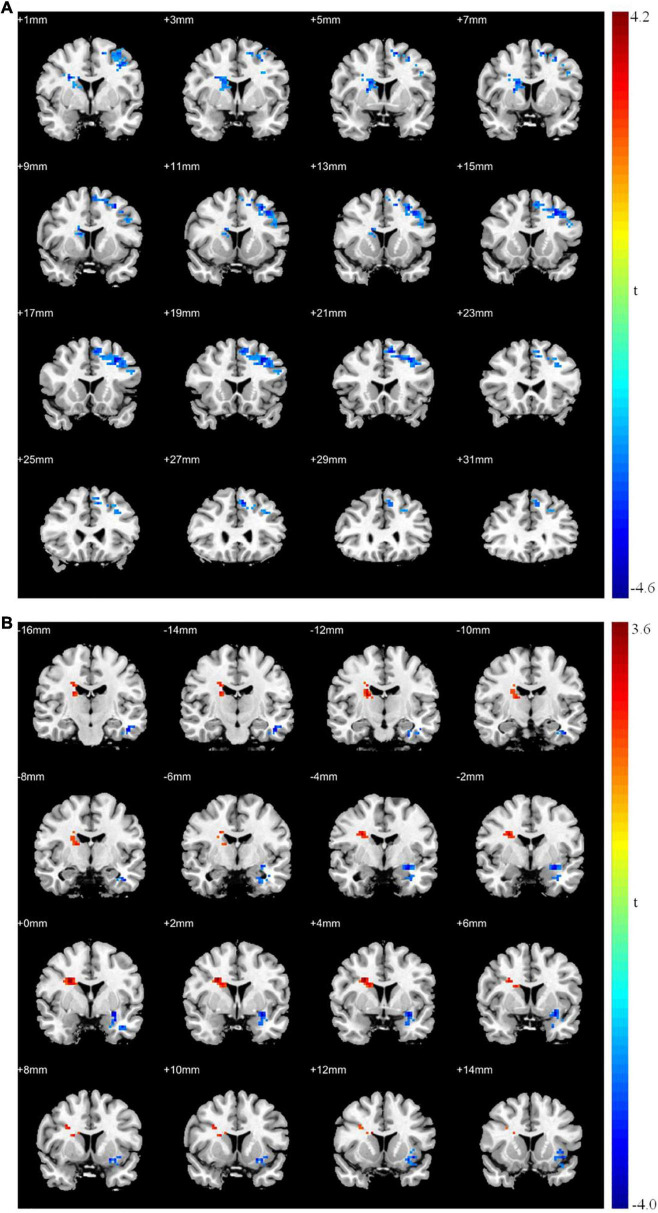
Granger causality analysis between patients with MDD and healthy controls. **(A)** Significant different brain regions from seed point (LMFG) to whole brain regions; **(B)** significant different brain regions from whole brain regions to seed point (LMFG). Red areas show brain regions where patients with MDD had increased causal connectivity than healthy controls; Blue areas show brain regions where patients with MDD had decreased causal connectivity than healthy controls. The color bar represents *t*-values.

The ALFF and ReHo analyses identified the left middle frontal gyrus as a brain region with synchronous changes; therefore, it was used as the seed point for GCA. The results showed that the causal connectivity from the seed point to the left superior frontal gyrus, right middle temporal gyrus, and right caudate nucleus was significantly weak (*p* < 0.05; Table 7 and Figure 3). Moreover, the causal connectivity from the left superior frontal, left inferior temporal, and left middle occipital gyri to the seed point was significantly weak (*p* < 0.05; Table 7 and Figure 3). However, the causal connectivity from the right caudate nucleus to the seed point was significantly strong (*p* < 0.05; Table 7 and Figure 3).

**TABLE 7:** Significant differences in Granger causality analysis between MDD and healthy controls.

Brain regions	Brodmann areas	Side	MNI coordinates			Cluster size	*t*-values
			*X*	*Y*	*Z*		
**x to y**							
MDD VS. healthy controls							
Superior frontal gyrus	46	Left	−30	18	39	345	−4.251
Middle temporal gyrus	21	Right	66	−24	0	95	−3.558
Caudate	48	Right	21	6	12	149	−3.964
**y to x**							
MDD VS. healthy controls							
Superior frontal gyrus	10	Left	−33	54	6	98	−3.258
Inferior temporal gyrus	20	Left	−48	−18	−30	145	−3.707
Middle occipital gyrus	19	Left	−33	−81	15	88	−3.284
Caudate	48	Right	24	0	27	98	3.514

**FIGURE 4:**
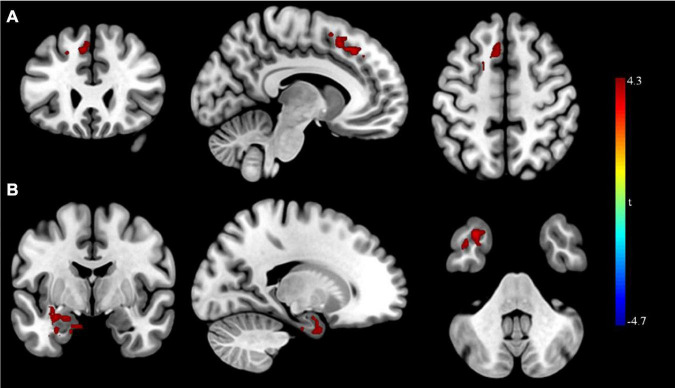
Granger causality analysis pre- and post- rTMS in MDD. **(A)** Brain regions showing group differences in causal connectivity from the seed point (LMFG) to whole brain in pre- and post-rTMS comparison; **(B)** Brain regions showing group differences in causal connectivity from the whole brain to seed point (LMFG) in pre- and post-rTMS comparison. Red areas show brain regions where post- rTMS patients with MDD had increased causal connectivity than pre- rTMS; Blue areas show brain regions where post- rTMS patients with MDD had decreased causal connectivity than pre-rTMS. The color bar represents *t*-values.

After rTMS treatment, the causal connectivity from the seed point to the superior frontal gyrus of patients with MDD was strengthened significantly (*p* < 0.05; Table 8 and Figure 4) and the causal connectivity from the left superior frontal and left inferior temporal gyri of patients with MDD to the seed point was also strengthened significantly (*p* < 0.05; Table 8 and Figure 4) compared with the causal connectivity before treatment.

**TABLE 8:** Significant differences in Granger causality analysis between pre- and post- rTMS.

Brain regions	Brodmann areas	Side	MNI coordinates			Cluster size	*t*-values
			*X*	*Y*	*Z*		
**x to y**							
Post-rTMS vs. pre- rTMS							
Superior frontal gyrus	46	Left	−33	18	39	129	4.081
**y to x**							
Post-rTMS vs. pre- rTMS							
Superior frontal gyrus	10	Left	−24	60	9	76	3.678
Inferior temporal gyrus	48	Left	−30	6	−12	246	3.583

### Correlation of Granger Causality Analysis Outcomes With HAMD-17 Score in Patients With Major Depressive Disorder After Repetitive Transcranial Magnetic Stimulation Treatment

**FIGURE 5:**
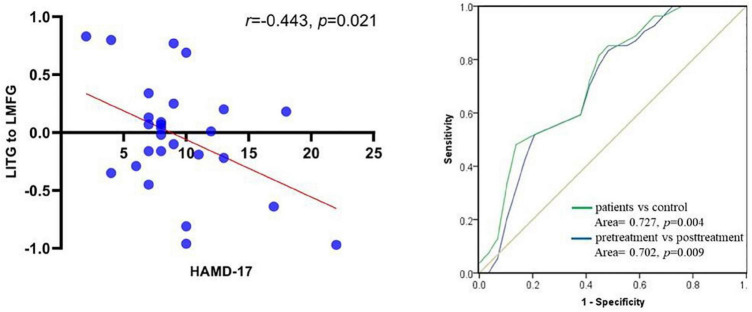
Scatter plot between causal connectivity from left inferior temporal gyrus to left middle frontal gyrus and score of HAMD-17 in post- rTMS of patients with MDD (left); ROC curve for causal connectivity from inferior temporal gyrus to seed point with patients between patients (pretreatment and posttreatment) and control (right).

The bidirectional causal connectivity between the left middle frontal gyri and left superior frontal gyri showed no significant negative correlation with the total HAMD-17 score (*p* > 0.05), whereas the causal connectivity from the left inferior temporal to the left middle frontal gyri showed a significant negative correlation with the total HAMD-17 score (*r* = −0.443, *p* = 0.021; Figure 5). The receiver operator characteristic (ROC) analysis was used to assess whether causal connectivity from inferior temporal gyrus to seed point can distinguish patients (pretreatment and posttreatment) and controls. The results showed that causal connectivity from inferior temporal gyrus to seed point successfully distinguished the patient group from the healthy control group and also pretreatment patients from the posttreatment patients (Figure 5).

## Discussion

In this study, the left dorsolateral prefrontal cortex was selected as the therapeutic target where the depression symptoms of patients with MDD were improved effectively. On the basis of ALFF and ReHo analyses, the ALFF and ReHo in the left superior frontal gyrus, left precuneus, and left middle frontal gyrus were significantly increased after rTMS treatment. GCA with the left middle frontal gyrus as the seed point revealed that after rTMS treatment, the bidirectional causal connectivity between the seed point and superior frontal gyrus was significantly improved and the causal connectivity from the inferior temporal gyrus to the seed point was also significantly improved and showed a remarkable negative correlation with the total HAMD-17 score. These findings demonstrate that the changes in the brain activity and causal connectivity in the frontal, parietal, and temporal regions may constitute the neural mechanisms underlying rTMS treatment in MDD.

As an important part of the prefrontal cortex (Li et al., 2013), the left superior frontal gyrus plays a vital role in self-awareness (Niendam et al., 2012) and negative emotion regulation (Chen et al., 2022), and it is involved in attention control and emotion regulation in patients with MDD (Dutta et al., 2014; Hamilton et al., 2015). In this study, we found that patients with MDD had significantly lower ALFF in the left superior frontal gyrus than healthy controls, which is consistent with the findings of previous studies (Liu et al., 2020; Zhang et al., 2021). The decreased spontaneous activities of brain neurons in the superior frontal gyrus may lead to low mood, anhedonia, and excessive negative self-evaluation in patients with MDD (Guo et al., 2013).

The left precuneus is a part of the superior parietal lobule (Zhu et al., 2018), which is involved in high-level cognitive functions such as episodic memory and self-reflection (Li et al., 2018). Studies have found that the abnormal neuronal activity in the precuneus of patients with MDD (Zhou et al., 2017) weakens the regulation of negative emotions (Zhong et al., 2016). The number of depressive episodes has a significant positive correlation with the ALFF in the precuneus (Jing et al., 2013). The present study demonstrated that patients with MDD had significantly higher ALFF in the left precuneus than healthy controls, indicating an excessive increase and abnormal activation of neuronal activities in this brain region of these patients; this finding is consistent with the that of previous studies (Guo et al., 2013; Yao et al., 2014; Zhang et al., 2021) and may be associated with a stronger negative self-awareness and sensory dysfunction (Gong et al., 2020; Wang et al., 2020).

The middle frontal gyrus is an important part of the ventromedial prefrontal cortex, which is involved in functions such as advanced cognition, autobiographical memory (van Heeringen et al., 2017), and emotion processing (Whalley et al., 2012) and cognition control dysfunction (Han et al., 2014). Evidence shows that an abnormal middle frontal gyrus leads to the dysregulation of top-down emotions and cognitive control disorder in patients with MDD (Lu et al., 2012). The ALFF in the middle frontal gyrus can not only specifically distinguish between the depressive episode and convalescence in MDD but also objectively reflect the severity of depression symptoms to a certain extent (Zhao et al., 2020). In the present study, the ALFF in the resting-state middle frontal gyrus was increased, further supporting the abnormal function of the middle frontal gyrus in patients with MDD. Abnormalities in functions such as abnormal emotional processing and cognitive memory may result from the compensatory increase in local neuronal activities in the middle frontal gyrus of patients with MDD (Guo et al., 2013). ReHo is also a sensitivity index of neuronal activity. The present study found that ReHo in the left middle frontal gyrus of patients with MDD was decreased, which is consistent with the finding of previous studies (Liu et al., 2021). The weakened synchronous and consistent activity of neurons in the middle frontal gyrus may lead to a decline in the ability to regulate emotions, thereby causing low mood with pessimistic and negative emotions in patients with MDD. In essence, abnormal ALFF and ReHo in the middle frontal gyrus reflect the disorders of local neuronal activity in patients with MDD in the resting state, which lead to the dysfunction of the middle frontal gyrus and dysregulation of emotional responses; these may represent important pathophysiological mechanisms of MDD (Geng et al., 2019).

High-frequency rTMS can regulate the excitability of the cerebral cortex (Xie et al., 2021). In this study, the left dorsolateral prefrontal cortex was targeted by high-frequency stimulation at 10 HZ, which directly enhanced the neural activity in the target-related brain region (Du et al., 2018). Therefore, an increase in the ALFF in the left superior frontal gyrus was observed in patients with MDD. The left superior frontal gyrus is the key brain region of the left dorsolateral prefrontal cortex. If it is damaged, the surrounding non-core organization brain regions compensate for the core brain region (Wang et al., 2019). The left middle frontal gyrus and left precuneus have a strong structural and functional connectivity with the left superior frontal gyrus (van den Heuvel and Sporns, 2011). If the function of the superior frontal gyrus is impaired, the middle frontal gyrus and precuneus may compensate for this impairment. Thus, an excessive increase in activity is observed in the left middle frontal gyrus and left precuneus. However, after treatment with high-frequency repetitive transcranial magnetic stimulation (HF-rTMS), the neural activity in the left superior frontal gyrus—the key brain region—was restored and the functions of the left middle frontal gyrus and left precuneus showed a tendency to be normal.

Our findings suggest that rTMS directly improves the neural activity in the target brain region stimulated indirectly improves the activity in local brain regions surrounding the target. The middle frontal gyrus is anatomically connected to the superior frontal gyrus through the uncinate fasciculus (Briggs et al., 2021), and this connection is involved in decision-making and emotional control. The functional connectivity between the middle and superior frontal gyri has been reported to be strengthened after antidepressant therapy (Porta-Casteràs et al., 2020). The findings of the present study are not only consistent with the findings of previous studies but also identify the direction of the causal connectivity between the middle and superior frontal gyri. The causal connectivity between the middle and superior frontal gyri was weak before treatment but strengthened after 15 days of continuous rTMS treatment; this suggests that the functional brain connectivity in patients with MDD tends to be normal and the negative thoughts can be improved, thus alleviating depression (Gröhn et al., 2019). Furthermore, the inferior temporal and middle frontal gyri are anatomically connected through the arcuate fasciculus, and these two also have a close functional connectivity, which is associated with emotional processing and episodic memory (Briggs et al., 2021). After rTMS treatment, the causal connectivity from the inferior temporal to the middle frontal gyri was strengthened and found to be negatively correlated with the total HAMD-17 score, indicating that the strengthening of such causal connectivity leads to the remission of depression symptoms.

## Conclusion

In this study, the ALFF, ReHo, and GCA were used to explore the mechanisms through which HF-rTMS improves depression symptoms in patients with MDD. Our findings indicate that the rTMS treatment was efficacious for reducing depressive symptoms. And the rTMS treatment could regulate the neuronal activity in frontal gyrus of patients with MDD; It further enhanced the connectivity among the frontal—temporal—parietal regions of the brain after rTMS treatment. These changes may represent the neural mechanism through HF-rTMS treatment and may be used as a clinical biomarker for the objective evaluation of the therapeutic efficacy of rTMS.

## Limitations

This study has some limitations. The sample size was small; this might have influenced the results and conclusion of this study. Moreover, Venlafaxine is the first of the SNRIs that provides dose-dependent norepinephrine reuptake inhibition; a dosage of 150 mg/day or higher is sufficient to produce noradrenergic activity, and it has low affinity for the postsynaptic receptors (Strawn et al., 2018; Zhou et al., 2021). A latest review has demonstrated that the brain function changes at least 4 weeks’ antidepressant pharmacotherapy in patients with MDD (Cattarinussi et al., 2021). In current study, venlafaxine, which is added from 75 to 150 mg/d within 15 days during rTMS treatment, may marginally affect brain function, but *post-hoc* test is difficult to control the confusion between drugs and rTMS treatment results. Future studies should ensure a larger sample size and enhance the experimental design to further discuss the therapeutic effects of rTMS in MDD.

## Data Availability Statement

The raw data supporting the conclusions of this article will be made available by the authors, without undue reservation.

## Ethics Statement

This study was reviewed and approved by the Medical Ethics Committee of Xijing Hospital (Approval document Number: KY20202055-F-1). The patients/participants provided their written informed consent to participate in this study.

## Author Contributions

MG, HW, and QT conceived and designed the experiments. MG, YS, ZM, YX, and ZW performed the experiments. MG, ZL, JL, and XG analyzed the data. HW and QT conceived the project and modified the manuscript. All authors read and approved the final manuscript.

## Conflict of Interest

The authors declare that the research was conducted in the absence of any commercial or financial relationships that could be construed as a potential conflict of interest.

## Publisher’s Note

All claims expressed in this article are solely those of the authors and do not necessarily represent those of their affiliated organizations, or those of the publisher, the editors and the reviewers. Any product that may be evaluated in this article, or claim that may be made by its manufacturer, is not guaranteed or endorsed by the publisher.

## Footnotes

1
https://rfmri.org/DPABIDiscussion

2
https://www.fil.ion.ucl.ac.uk/spm/software/spm12/
